# Role of Herpes Simplex Envelope Glycoprotein B and Toll-Like Receptor 2 in Ocular Inflammation: An Ex Vivo Organotypic Rabbit Corneal Model

**DOI:** 10.3390/v11090819

**Published:** 2019-09-04

**Authors:** Andreana Marino, Simona Pergolizzi, Francesco Cimino, Eugenia Rita Lauriano, Antonio Speciale, Valeria D’Angelo, Mariaconcetta Sicurella, Rafaela Argnani, Roberto Manservigi, Peggy Marconi

**Affiliations:** 1 Department of Chemical, Biological, Pharmaceutical and Environmental Sciences, University of Messina, Polo Annunziata, 98168 Messina, Italy; 2 Department of Chemical and Pharmaceutical Sciences (DipSCF), University of Ferrara, Via Fossato di Mortara 64/A, 44121 Ferrara, Italy; 3 Department of Life Sciences and Biotechnology (SVeB), University of Ferrara, Via Luigi Borsari 46, 44121 Ferrara, Italy

**Keywords:** HSV-1 gB, TLR2, ex vivo corneal model, mydriasis

## Abstract

It has been recently reported, using in vitro studies, that the herpes simplex virus 1 (HSV-1) encoded envelope glycoprotein B (gB1) interacts with cell surface toll-like receptor 2 (TLR2) and induces the secretion of interleukin-8 (IL8), a representative marker of inflammatory cytokine activation. The purpose of this study is to investigate the role of gB1 in activating host inflammatory responses by using a secreted form of gB1 (gB1s) and an ex vivo organotypic rabbit corneal model. Abraded corneas exposed to gB1s alone or to the recombinant protein mixed with anti gB polyclonal antibody were cultured in an air–liquid interface. The corneas exposed to gB1s show the appearance of mydriasis and high levels of TLR2 and IL-8 mRNAs transcripts were detected in the superficial layer of corneal epithelial cells. Histological stain and immunohistochemical analyses revealed morphological changes in the epithelium of the treated corneas and variations in expression and localization of TLR2. Collectively these findings provide new insight into the pathogenesis of HSV-1 ocular infection by demonstrating the leading role of gB in activating an inflammatory response and in the appearance of mydriasis, a sign of HSV-1 anterior uveitis.

## 1. Introduction

According to WHO’s global estimates, the herpes simplex virus type 1 (HSV-1) has infected over 3.7 billion people under the age of 50 (67% of the world population) and is responsible for the vast majority of ocular infections [1,2]. HSV-1 is associated with numerous ocular diseases, including herpetic epithelial keratitis (HEK) and herpetic stromal keratitis (HSK) [3,4,5]. HEK progresses to involve the stroma in approximately 20% of cases. The global incidence of HSV keratitis is about 1.5 million, with 40,000 new cases of monocular visual impairment or blindness per year [1]. HSK is the result of an intense inflammatory response to HSV invasion of the anterior stroma, either through the reactivation of latent HSV in sensory nerves or to the direct spread from epithelial infection. This stromal inflammation may be diffuse, multifocal, or focal, and can be associated with up to 9% of cases of anterior uveitis [6]. HSV-induced anterior uveitis is a combination of viral inflammatory infection and antiviral responses that in the absence of corneal disease often remains undiagnosed, resulting in inadequate treatment [4]. Pathological findings in HSV ocular disease are due to both direct viral effects and host innate and adaptive immune responses [7]. The expression of toll-like receptors (TLRs) has been reported in most ocular tissues, such as conjunctiva, cornea, stroma, uvea, sclera, and retina. These receptors play an important role in triggering the earliest immune response, leading to inflammation [7,8]. Several studies indicate a positive role played by TLRs in the clearance of HSV-1 from the cornea during primary infection, but they are also associated with eye disease immunopathology [9,10].

HSV infects corneal cells with the same mechanism encountered in other tissues. It enters through fusion with the plasma membrane or via endosomes through an orchestrated process that requires gB, the most ubiquitous envelope glycoprotein in human herpesviruses, and three other essential envelope glycoproteins (gD and gH/gL), activated in a cascade fashion [11,12,13,14]. Viral entry is initiated by the interaction of gB and the non-essential glycoprotein C with heparan sulfate proteoglycan (HSPG). The glycoprotein gB regulates a process called “surfing”, where viral particles travel along filopodia to reach the cell surface, and mobilize lipid rafts, where gB receptors or co-receptors are localized [15]. After the initial attachment, gD is needed for receptor-mediated endocytosis or for the direct fusion of the viral envelope to the plasma membrane [14]. Therefore, following the interaction between gD and its receptors, a conformational change occurs that transmits an activation signal to recruit the fusion complex that comprises the trimeric gB, gH, and gL which mediates fusion with the host cell membrane, the final step in viral entry [11,14,16]. It is worth stressing that gB was the first HSV envelope glycoprotein identified to induce cell fusion [17].

Compton and co-workers initially identified TLR2 as a cellular factor that mediates the innate immune response to human cytomegalovirus (HCMV) infection [18] and demonstrated that HCMV gB plays a role in activating TLR2 in permissive human fibroblast cells [19]. Recent in vitro studies, using a truncated and soluble form of HSV-1 gB (gB_730t-st_), have shown that also HSV-gB1 is a ligand to TLR2 [20,21]. It was reported that gB1 binds TLR2 and activates NF-kappaB through the MyD88/TRAF6-dependent signaling pathway [20]. TLR2 recognition of gB is hypothesized to play a role in the immunopathology of HSV infection, but no ex vivo or in vivo models have been yet developed that associate this interaction with specific HSV diseases. The role of gB1 in ocular disease, leading to increased inflammation and subsequent development of inflammation-associated lesions, is still not understood due to the complexity of the interaction with the other viral proteins in HSV infection [10]. In the present study, by using a constitutively secreted form of gB1 (gB1s) [22], we developed an ex vivo rabbit organotypic corneal model [23]. The ex vivo model was selected as the first step to study the effect of gB1 in the ocular environment, as cell cultures fail to mimic corneal multilayer epithelium, while in vivo studies require the use of a large number of animals, which are expensive and required elaborate maintenance procedures. Furthermore, the welfare of these animals has become an important ethical issue leading to the promotion of the attitude of “replacement, reduction and refinement” in the use of animals in research, as stated in the EU/Directive 2010/63. Treating the corneal epithelium with gB1s we were able to provide evidence that this glycoprotein induces the expression of TLR2 and IL8 mRNAs, with clear signs of inflammation, associated with morphological changes in the epithelial architecture. Furthermore, the treatment with gB1s induces the appearance of mydriasis (pupillary dilation), a clinical sign of HSV-1 anterior uveitis [24,25,26]. These results are the first evidence that gB1, present on the viral envelope, plays a key role in modulating TLR2 expression and has a clear effect on the ocular inflammation and immunopathology.

## 2. Materials and Methods

### 2.1. Cells and Viruses

Vero cells (African green monkey epithelial cells, ATCC CCL-81) and HEK293 gB1s (293 human embryonic kidney cells constitutively secreting gB1s, generated in our laboratory), were maintained at 37 °C in Dulbecco’s modified minimal essential medium (DMEM, Euroclone, Italy) supplemented with 10% fetal bovine serum (FBS). KOS wild type HSV-1 and KOS gBpK^−^ mutant virus, deleted for the HSPG binding-lysine-rich (pK^−^ minus) sequences of gB, were produced and titrated as previously described [27,28].

### 2.2. Antibodies

The anti-gB monoclonal antibody (mAb) (Ig2a, clone I-144), used to purify the gB1s, was a gift from Dr. Patricia Spear.

Anti-gB1s and anti-pK rabbit polyclonal antibodies (pAbs), for gB1s neutralization, were developed and characterized in our laboratory [29,30]. In particular, a 13 aa gB1 sequence, named DTK, comprising the 9 aa pK domain was used to immunize rabbits and generate the anti-pK pAb [30].

### 2.3. gB1s Production and Neutralization

gB1s was constructed by excision of 639 nucleotide fragment, which encodes for the transmembrane anchor, from a gB1 gene of HSV-1 F strain. The coding sequences for the extramembrane and the C-terminal intracytoplasmic regions were reconstructed by in-frame self-ligation into a pRP-RSV plasmid under the control of the Rous sarcoma virus as previously described [22]. The pRP-RSVgB1s plasmid was stably transfected in HEK293 cells and cellular clones expressing gB1s were isolated, as previously described [22,29]. Briefly, human 293 cells constitutively expressing gB1s were maintained at 37 °C and grown at confluence in Dulbecco’s modified Eagle’s medium (DMEM) supplemented with 10% FBS. The growth medium was removed and replaced with serum-free culture medium for 24 h. The amount of gB1s in the crude culture medium, determined by densitometric analysis of polyacrylamide gel, was about 0.20 μg/mL per 10^6^ cells. The secreted product was identified in both WB and ELISA, concentrated by ultrafiltration and immunoaffinity-purified by using I-144 mAb.

gB1s neutralization was performed, as previously reported [30], by incubating the recombinant protein, at a concentration of 1250 ng/50 μL, for 3 h at 37 °C, with 50 μL of anti-pK pAb; while the same concentration of gB1s was incubated with 30 μL of anti-gB1s pAb added with additional 20 μL of PBS, to a final volume of 100 μL. The mixtures, containing 125 ng of gB1s in a final volume of 10 μL, were used to treat selected samples of corneal cultures, as described below.

### 2.4. Experimental Design

Normal rabbit eyes, obtained from a local abattoir, were washed with phosphate-buffered saline buffer (PBS) and maintained in DMEM/Ham’s F-12, (Euroclone, Italy) with antibiotic/antimycotic solution (1:200). To prepare the corneal culture, the sclera-corneal ring of eighteen eyes were placed on the organ support in dishes containing the tissue culture medium, as previously reported [23,31,32]. A demarcated central area (5 mm) of the epithelial layer of the cornea was abraded using a surgical trephine. The sclera-corneal rings were randomly divided into six groups of three rings each and the abraded corneas were treated as follows: group 1, received 125 ng of gB1 in 10 µL of PBS; group 2, a mixture of gB1s/anti-gB1s pAb; group 3, a mixture of gB1s/anti pK pAb; and the control groups 4, 5, and 6 received 10 µL of PBS, bovine serum albumin (BSA) and pre-immune (pI) rabbit serum, respectively. The organ cultures were incubated at 37 °C in a humidified atmosphere of 6% CO_2_ for 48 h. The culture medium, in the dishes, was changed once during the experiment (after 24 h). All experiments were performed in triplicate and repeated three times.

### 2.5. Quantitative RT-PCR

Quantitative RT-PCR experiments to detect TLR2 and IL8 mRNA were performed as described [23,32]. Briefly, the superficial layers of the epithelium were removed by using a 13 mm pre-autoclaved membrane filter. Total RNA from the epithelium was extracted using RNeasy Mini Kit (Qiagen) following the supplier’s instructions. PCR amplification was performed with an Applied Biosystem 7300 Real-Time PCR System, (Monza, Italy) coupled with the SYBR^®^ green JumpStart™ Taq Ready Mix kit using specific primers for TLR2, GAPDH, and IL-8 at optimized concentrations and cycling conditions. GAPDH was used as housekeeping gene for normalization. The fold increase was compared with the cells of intact corneas not exposed to gB and mRNA expression was determined using the 2^−ΔΔCt^ method [33]. All experiments were performed in triplicate and repeated three times. Results were expressed as means ± SD and statistically analyzed by a one-way ANOVA test, followed by Tukey’s HSD. Differences in groups and treatments were considered significant for *p* < 0.05.

### 2.6. Histology—Light Microscopy

Cultured rabbit cornea samples were fixed in 4% paraformaldehyde (Immunofix^®^, BIO-OPTICA Milano, Italy) in 0.1 M phosphate-buffered saline (PBS, pH 7.4) for 4 h at 4 °C. The samples were washed with a solution of phosphate buffer 0.1 M for 1 h, dehydrated in graded ethanol (30°–100°), cleared in xylene, and finally embedded in Paraplast^®^ (McCormick ScientificLLC, St. Louis, MO, USA). Serial sections (5-µm thick), were obtained by a rotary microtome (LEIKA 2065 Supercut) and were stained with the histological stain Hematoxylin-Eosin (H/E) method [32]. All samples were observed and photographed with an optical microscope Axioshop, Zeiss, equipped with a Sony^®^ DSC-85 camera.

### 2.7. Immunofluorescence

Serial sections were deparaffinized and rehydrated, rinsed several times in PBS, and blocked in 10% normal goat serum for 1 h. TLR2 rabbit antibody (a mixture of synthetic peptides corresponding to amino acid residue(s) 180e196, 353e370, and 473e489 of Human TLR2) (Active Motif, Vinci-Biochem, Florence, Italy), was diluted in a permeabilizing solution (PBS, 0.2% Triton X-100, 0.1% sodium azide) according to the optimal dilutions and placed on the slides to incubate overnight at room temperature. TLR2 localization was carried out as previously reported by Lauriano et al. [34,35]. The sections were then treated with fluorescent-labeled secondary antibody diluted in PBS, (goat-anti-rabbit Alexa Fluor 594 donkey anti-rabbit IgG TRITC conjugated) (MolecularProbes, Invitrogen, Eugene, OR, USA), and left to incubate at room temperature for 2 h in the dark. After washing, the sections were mounted with Vectashield (Vector Labs, Burlingame, CA, USA) to prevent photobleaching and coverslipping. Control experiments were performed excluding primary antibody.

### 2.8. Laser Confocal Immunofluorescence

Sections were analyzed and images acquired using a Zeiss LSMDUO confocal laser-scanning microscope with META module (Carl Zeiss MicroImaging GmbH, Germany). Each image was rapidly acquired in order to minimize photo-degradation. Digital images were cropped and the figure montage prepared using Adobe Photoshop7.0 (Adobe Systems, San Jose, CA, USA).

## 3. Results

### 3.1. Characterization of Polyclonal Antibodies against gB1s and Anti gB1-pK Sequences

**Figure 1:**
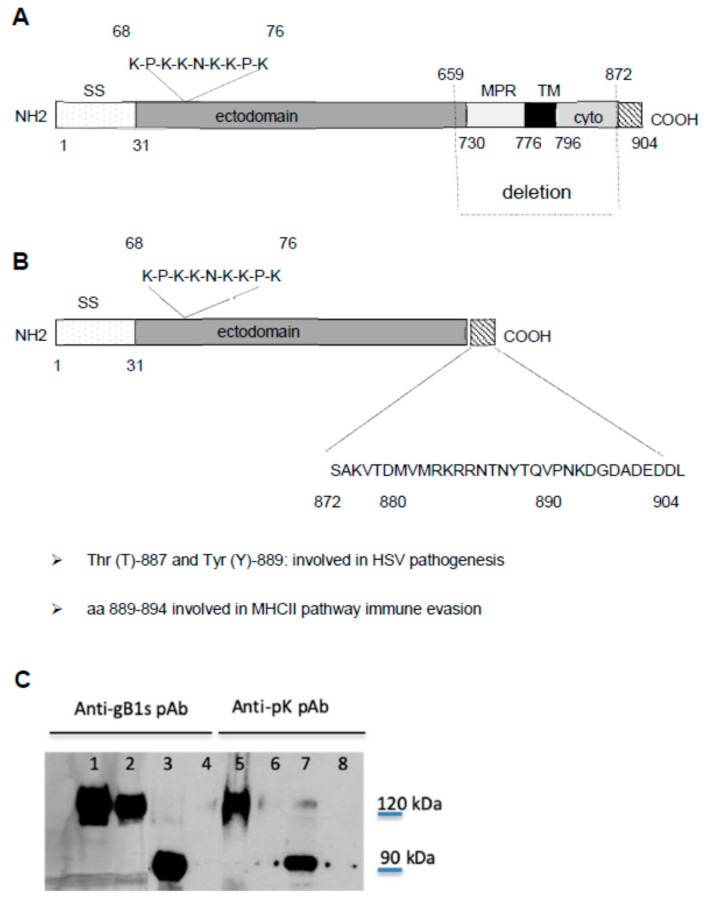
Schematic representation of KOS gB1 (**A**) and gB1s (**B**) and western blot analysis using anti-gB1s and anti-pK pAbs (**C**). SS, signal sequence; MPR, membrane-proximal region; TM, trans-membrane region; ectodomain and (cyto) cytodomain regions. Schematic representation of (**A**) full-length gB1 and (**B**) gB1s secreted protein obtained from the gB1 gene encoding the MPR and the TM regions, after having deleted 639 nucleotides and reconstructed the gene with the ectodomain and COOH cytodomain. The sequence from 872 to 904 represents the terminal sequence of the cytodomain present in gB1s. Sequence 68 to 76 is the lysine-rich region (pK) that binds HSPG. (**C**) WB analysis of gB1s recombinant protein (≈90 kD) using the anti-gB1s and anti-pK pAb (lane 3 and 7) and, as controls, full length KOS wt gB1 (120 kDa) (lane 1 and 5) or gBpK^−^ mutant (lane 2 and 6) using the anti-gB1s and anti-pK pAbs respectively and mock uninfected cells tested with anti-gB1s and anti-pK pAbs (lanes 4 and 8). The anti-pK pAb is not able to recognize the KOS gBpK^−^ mutant.

In our study we used a recombinant soluble form of gB1 (gB1s), expressed constitutively in 293 cells, that was engineered and reconstructed with the COOH terminal intracytoplasmic domain [22] (Figure 1). This recombinant protein has been shown to possess most of the biological properties of the wild type gB1 [36,37,38,39]. A schematic representation of full-length gB1 and gB1s is shown in Figure 1A,B, respectively. Both glycoproteins contain the HSPG pK-binding domain of gB, that is positioned within amino acid residues 68 to 76 and has the pK sequence KPKKNKKPK. To test the specificity of the antibodies used in our experiments, purified gB1s and lysates from infected cells with HSV-1 KOS and recombinant KOS deleted in the pK sequence (gBpK^−^), were analyzed by Western blotting using the anti-pK pAb and the anti-gB1s pAb. As Figure 1C shows, both the antibodies, gB1s pAb and anti-pK pAb, were able to react with gB1s (lanes 3 and 7) respectively. Moreover, the anti-pK pAb reacts with the full-length gB1 in cells infected with KOS (lane 5), but it does not recognize gB1, deleted in pK sequence, in cells infected with gBpK^−^ mutant (lane 6). It is important to underline that neutralization studies, conducted in our laboratories, have demonstrated that anti-pK and anti-gB1s polyclonal antibodies can inhibit HSV infection, indicating that these antibodies recognize native gB1 protein [29,30].

### 3.2. Biological Activity of gB1s in an ex-vivo Rabbit Corneal Model

#### 3.2.1. Mydriasis in gB1s Treated Corneas

**Figure 2:**
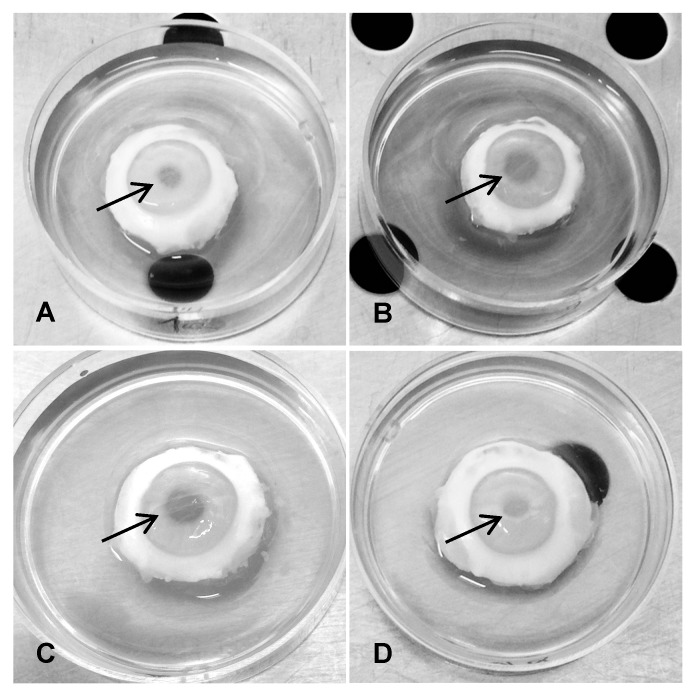
Clinical signs in treated corneas. The sclero-corneal rings were randomly divided into groups of three rings each treated as follows: (**A**), pI (pre-immune) rabbit serum as control; (**B**), gB1s recombinant protein; (**C**), gB1s recombinant protein preincubated with the anti-pK rabbit polyclonal specific antibody; (**D**), gB1s recombinant protein preincubated with the anti-gB1s rabbit polyclonal specific antibody. Arrows are indicating the pupil. For each group *n* = 3.

In order to investigate the biological activity of HSV-1 gB1s, experiments were performed in an ocular ex vivo rabbit model that mimics human corneal multilayer epithelium [40]. Rabbits represent the ideal animal model to study ocular HSV-1 infection, since it shares many characteristics with the naturally occurring infections in humans. Figure 2 shows four of six groups of sclero-corneal rings of rabbit eyes treated with: PBS, BSA, and preimmune (pI) rabbit serum as controls; gB1s recombinant protein; gB1s recombinant protein preincubated with anti-pK pAb and recombinant protein preincubated with anti-gB1s pAb. The figure reports only one of the three control groups as all of them show the same biological behavior. The results demonstrate that the corneas exposed to gB1s alone and gB1s preincubated with anti-pK pAb developed mydriasis with a mean difference in pupil size of about 2.9 mm with respect to all other groups (Figure 2B,C), whereas the corneas exposed to pI serum control or to gB1s preincubated with anti-gB1s pAb did not show mydriasis (Figure 2A,D). These results demonstrated that the anti-gB1s pAb was able to neutralize the biological effect of gB1s (Figure 2D), while no effect was seen with the viral glycoprotein treated with anti-pK pAb (Figure 2C). This observation seems to rule out the hypothesis that the gB1 pK region, which acts as one of the ligands for the cellular HSPG receptor, is involved in the appearance of pupil dilatation.

#### 3.2.2. TLR2 and IL8 mRNA Expression in Superficial Layers of Corneal Epithelial Cells Exposed to gB1s

**Figure 3:**
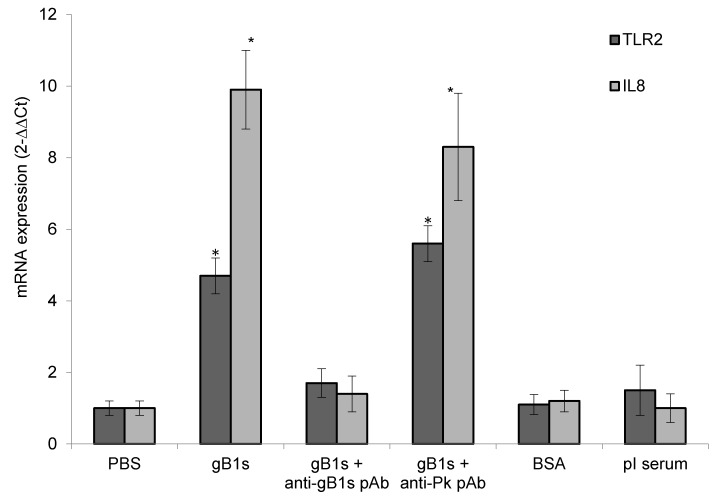
TLR2 and IL8 mRNA expression. Corneal epithelial cells were treated with 125 ng of gB1s alone or gB1s pre-treated with anti-pK pAb or gB1s pre-treated with anti-gB1s pAb or PBS, BSA, and pre-immune (pI) rabbit serum as control samples. For each group *n* = 3. Gene expression was expressed as 2^−^^ΔΔCt^ normalized to PBS control. Results were expressed as means ± SD from three different experiments and statistically analyzed by a one-way ANOVA test, followed by Tukey’s HSD. Differences in groups and treatments were considered significant for *p* <0.05. * *p* < 0.05 vs PBS control.

To investigate the expression and function of TLR2 induced by HSV-1 gB1s protein, RNA was extracted from the superficial layers of corneal epithelial cells of each group. TLR2 and IL8 mRNA levels were evaluated by Real-Time PCR. The presence of IL8 mRNA was used as a representative marker of inflammatory cytokines activation [20]. Our results demonstrate that TLR2 mRNA (4.7 ± 0.5) and IL-8 mRNA (9.9 ± 1.1) levels were significantly higher in epithelial cells exposed to gB1s with respect to levels of control cells (1.0 ± 0.2) (Figure 3). Interestingly, the preincubation of gB1s with anti-gB1s pAb resulted in the reduced expression of TLR2 mRNA (1.8 ± 0.2) and IL-8 mRNA (1.6 ± 0.3), similar to that observed in the three control groups (Figure 3). On the contrary, the pre-incubation of gB1s with anti-pK pAb did not affect the level of TLR2 mRNA (5.6 ± 0.5) and IL-8 mRNA (8.3 ± 1.5), that was similar to that observed in samples treated with gB1s alone. These results clearly show that, in our ex vivo model, gB1s is able to enhance the expression of TLR2 and IL-8 mRNAs, compared to the controls. These biological effects disappeared when gB1s was treated with neutralizing anti-gB1s pAb, whereas the treatment with anti-pK pAb did not seem to have any effect. These data suggest that gB1s stimulates TLR2 and IL8 transcript accumulation, independently from the HSPG pK binding domain.

#### 3.2.3. Morphological Changes in Sections of Rabbit Corneal Epithelium and Expression of TLR2 on Cell Surface

**Figure 4:**
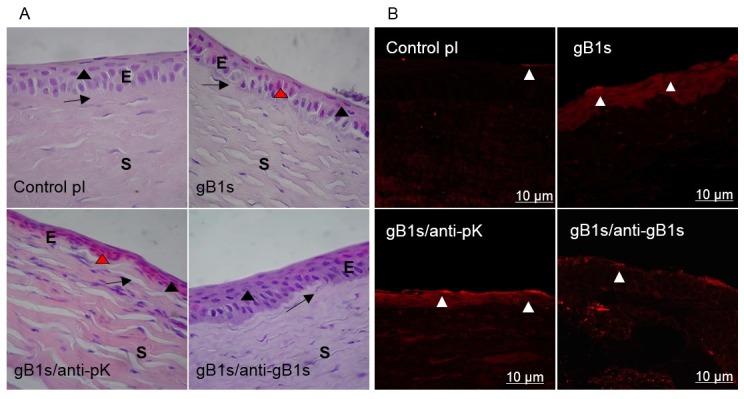
Morphological observations (**A**) and expression of TLR2 on the cell surface (**B**) in sections of rabbit corneal epithelium samples. (**A**) Hematoxylin-Eosin (H/E) stain. Epithelium (E), Stroma (S). The suprabasal layer (black arrowhead) and the basal layer (arrow) are indicated. Magnification 100×. control; pre-immune (pI) rabbit serum as control sample; epithelial cells are columnar in basal layer while wing and surface cells are flattened. gB1s; in cornea treated with recombinant protein, epithelial cells are flattened, disarranged in all layers, some cells present Cowdry bodies (red arrowhead). gB1s/anti-pK; in cornea treated with recombinant protein preincubated with anti-pK gB1s pAb, epithelial cells are flattened and strongly eosin stained. gB1s/anti-gB1s; with recombinant protein preincubated with anti-gB1s pAb, cells of the basal layer became columnar and in the suprabasal layer are flattened (as in the control in pI). (**B**) Immunofluorescence staining with anti-TLR2 Ab on abraded cornea exposed to: control pI; pre-immune rabbit serum as control sample; gB1s recombinant protein; gB1s/anti-pK, gB1s preincubated with anti-pK gB1s pAb; gB1s/anti-gB1s, gB1s preincubated with anti-gB1s pAb.

Morphological changes in abraded treated corneas were examined by histological stain with hematoxylin-eosin (Figure 4A) and changes in TLR2 expression and localization were observed by immunohistochemical analyses (Figure 4B). As is shown in Figure 4A, the pI control sample maintains the corneal architecture where the basal cells were columnar with rounded heads, flat bases, and oval nuclei elongated along the cell’s long axis; the second epithelial cell layer was characterized by wing cells, polyhedral, anteriorly convex forming a cap over basal cells and by keratinocytes, superficial squamous cells, with flattened nuclei. In the corneal stroma, the lamellae were arranged in layers parallel to each other and with the corneal surface. In Figure 4B, the pI control sample shows a weakly detectable TLR2 expression on the membranes of the epithelial cells and in the corneal stroma. In samples exposed to PBS and BSA the epithelial cells showed the same morphology present in the pI control group (data not shown).

In samples treated with gB1s (Figure 4A), the morphology of the epithelium was radically altered, with signs of inflammation. The basal cells, as well as those of the upper layers, appear flattened. Some cells showed intranuclear eosinophilic bodies, typical of HSV infection (Cowdry type A inclusions) [41]. Moreover, the stromal lamellae were not arranged in a parallel pattern and large colored nuclei were detectable. In Figure 4B, TLR2 was strongly positive in gB1s treated samples, both on the cell surface and within the cytoplasm. Interestingly, in the corneal stroma, immunofluorescence showed that keratocytes, differentiated into myofibroblasts, express TLR2 (Figure 4B).

Samples exposed to the gB1s/anti-pK pAb mixture (Figure 4A) exhibited signs of inflammation even more intense than those exposed only to gB1s. Morphological alterations were clearly evident, with strongly eosin-stained epithelial cells flattened in all layers. The corneal stroma showed eosin-stained, thickened fiber bundles, and more keratocytes/myofibroblasts activated with rounded nuclei [23] (Figure 4A). Immunolocalization in these samples showed epithelial cells strongly positive to TLR2 both on the surface and in the cytoplasm (Figure 4B). The corneal stroma showed a large number of cells expressing TLR2 (Figure 4B).

In samples exposed to the gB1s/anti-gB1s pAb mixture, the recovery of epithelial morphology was evident in that the cells of the basal layer became columnar, whereas the cells in the suprabasal layers were more flattened, similar to those in the control group (Figure 4A). We noticed occasional eosinophilic cells in the epithelium. In the stroma, the lamellae were distributed in a regular pattern and the keratocyte nuclei appeared still numerous and flattened. Immunolocalization for TLR2 in these specimens revealed that the antigen was restricted to the plasma membrane of the epithelial cells like in the control samples (Figure 4B).

## 4. Discussion

Two recent in vitro studies, have demonstrated that HSV-1-encoded gB interacts with TLR2 [20,21], releasing IL8 cytokine from human THP-1 monocytes [20]. However, since the appearance of these works no, ex vivo or in vivo, studies have been conducted to understand the pathological effect due to this interaction.

In this work, we used an ex vivo rabbit organotypic corneal model [23,32] and a secreted form of HSV-1 gB [22] to test the role of this glycoprotein in ocular inflammation [40,42]. In particular, the ex vivo rabbit organotypic tissue represents the more adaptable model for our study because it (a) maintains the corneal architecture, (b) allows interaction between the different cell type in the cornea, (c) includes iris pigment epithelial cells (IPE) that respond to PAMPS through the activation of TLRs, particularly TLR2 [8], and (d) permits the study of molecular sensors of innate immunity. However, our experimental approach is not intended to be a model of HSV eye infection, in which latency, reactivation, and immune response are significant components of the pathogenesis.

In our study, we have utilized the gB1s developed in our laboratory with unique characteristics: (a) to be constitutively expressed in human cells that allows easy production and purification and (b) to preserve the COOH terminal intracytoplasmic domain, which differentiates it from the previously reported soluble truncated form of gB (gB_730-st_) that lacks the COOH terminus [20,21]. It is highlighting that the two motifs in the cytoplasmic tail of gB, tyrosine at position 889 (Tyr-889) and at position 887 (Thr-887), affect neurovirulence and pathogenesis in the mouse model [43], while the cytosolic gB1 sequence 889–894 impacts on the major histocompatibility complex (MHCII) pathway for immune evasion [44].

Our data show that the corneal epithelium exposed to gB1s significantly induces and enhances the expression of TLR2 and IL8 mRNAs. Furthermore, a marked clinically significant mydriasis appears. Our results not only revealed mydriasis and overexpression of TLR2 and IL8 but correlate gB1 with important and detailed morphological changes in corneal architecture with signs of inflammation. These observations were corroborated by our experimental data where the pre-treatment of gB1s with a neutralizing antibody, prior to exposure to the corneal epithelium, reduces TLR2 and IL8 expression to baseline levels, with the recovery of epithelial morphology and absence of mydriasis.

Our attempt to explain the appearance of mydriasis generated two hypotheses. The first suggests that gB1s interferes with the control of pupillary dilation by modulating the expression of TLR2 present in uveal epithelium. In particular, TLR2 and gB1 may have a critical role in the pathogenesis of uveitis, which is associated with high levels of IL-8 in the aqueous humor [45]. In fact, among the numerous ocular diseases caused by HSV, mydriasis is present only in clinical cases of herpetic uveitis, in the absence of pupil dilating medications [24,25,26]. The second hypothesis links the application of gB1s in the scarred cornea with changes in the efflux of calcium. It has been reported that HSV infection is regulated by an early cellular Ca^2+^ response that requires the concerted activity of gB, gD, and gH/gL heterooligomers. Specifically, HSV triggers three calcium responses to facilitate viral entry into cells. First, engagement of HSPG by gB induces an initial transient depletion of calcium [46]. Secondly, the interaction of gD with nectin 1 receptor results in the release of plasma membrane Ca^2+^ stores [13,46]. Lastly, the completion of the fusion and penetration process, involving gD, gH/gL, and gB, requires the release of IP_3_R sensitive intracellular Ca^2+^ stores [12,13]. As it appears from this cascade of events, in the initial binding of HSV to the cell surface, gB acts as an antagonist of Ca^2+^ release. At this regard, it is noteworthy to observe that mydriasis often appears following the treatment with calcium antagonist in clinical treatments [47].

In an effort to identify the domain of gB1 that recognizes and/or regulates TLR2 we started from the reported evidence that in human immunodeficiency 1 virus (HIV-1) infection gp120-TLR2 interaction requires the presence of HSPG to activate pro-inflammatory pathways [48]. On the basis of this observation we pre-treated gB1s with neutralizing antibody against the pK region of gB1 [30], which represents one of the HSPG binding domains of the glycoprotein [27]. In contrast to the HIV-1 model [48], the pre-treatment of gB1s with anti-pK pAb had no effect on reducing the proinflammatory response caused by gB1s, suggesting that the pK domain of gB1 to HSPG is not required for TLR2 stimulation. Further studies are needed to evaluate whether other co-receptors of gB1 are involved in TLR2 regulation or to find which gB1 domain is involved in TLR2 activation.

In summary, our work provides the basis for a deeper understanding of ocular immunopathology following HSV-1 infection since the cornea is directly involved in the immune response through the expression of proinflammatory genes, release of inflammatory cytokines, and recruitment of inflammatory cells. This work is the first evidence that indicates an important role played by gB1 and TLR2 in viral pathogenesis and, in particular, in the proinflammatory phase of HSV ocular infection. Therefore, significant future work will be done to understand the critical structure-function of gB related to cellular and tissue damage via inflammation.

Accordingly, the use of HSV-gB1-specific monoclonal antibodies [49] and strategies to inhibit gB-TLR2 interactions could be effective approaches in the treatment of HSV-induced ocular diseases [50,51] by selectively modulating and dampening the inflammatory stimulus.

## References

[1] Farooq A.V., Shukla D. (2012). Herpes simplex epithelial and stromal keratitis: An epidemiologic update. Surv. Ophthalmol..

[2] Looker K.J., Magaret A.S., May M.T., Turner K.M., Vickerman P., Gottlieb S.L., Newman L.M. (2015). Global and Regional Estimates of Prevalent and Incident Herpes Simplex Virus Type 1 Infections in 2012. PLoS ONE.

[3] Rowe A.M., St Leger A.J., Jeon S., Dhaliwal D.K., Knickelbein J.E., Hendricks R.L. (2013). Herpes keratitis. Prog. Retin. Eye Res..

[4] Tsatsos M., MacGregor C., Athanasiadis I., Moschos M.M., Hossain P., Anderson D. (2017). Herpes simplex virus keratitis: An update of the pathogenesis and current treatment with oral and topical antiviral agents-response. Clin. Exp. Ophthalmol..

[5] Hlinomazova Z., Loukotova V., Horackova M., Sery O. (2012). The treatment of HSV1 ocular infections using quantitative real-time PCR results. Acta Ophthalmol..

[6] Teitelbaum C.S., Streeten B.W., Dawson C.R. (1987). Histopathology of herpes simplex virus keratouveitis. Curr. Eye Res..

[7] Lambiase A., Micera A., Sacchetti M., Mantelli F., Bonini S. (2011). Toll-like receptors in ocular surface diseases: Overview and new findings. Clin. Sci..

[8] Mai K., Chui J.J., Di Girolamo N., McCluskey P.J., Wakefield D. (2014). Role of toll-like receptors in human iris pigment epithelial cells and their response to pathogen-associated molecular patterns. J. Inflamm..

[9] Ma Y., He B. (2014). Recognition of herpes simplex viruses: Toll-like receptors and beyond. J. Mol. Biol..

[10] Sarangi P.P., Kim B., Kurt-Jones E., Rouse B.T. (2007). Innate recognition network driving herpes simplex virus-induced corneal immunopathology: Role of the toll pathway in early inflammatory events in stromal keratitis. J. Virol..

[11] Agelidis A.M., Shukla D. (2015). Cell entry mechanisms of HSV: What we have learned in recent years. Future Virol..

[12] Campadelli-Fiume G., Menotti L., Avitabile E., Gianni T. (2012). Viral and cellular contributions to herpes simplex virus entry into the cell. Curr. Opin. Virol..

[13] Cheshenko N., Trepanier J.B., Stefanidou M., Buckley N., Gonzalez P., Jacobs W., Herold B.C. (2013). HSV activates Akt to trigger calcium release and promote viral entry: Novel candidate target for treatment and suppression. Faseb J. Off. Publ. Fed. Am. Soc. Exp. Biol..

[14] Koujah L., Suryawanshi R.K., Shukla D. (2019). Pathological processes activated by herpes simplex virus-1 (HSV-1) infection in the cornea. Cell Mol. Life Sci..

[15] Tiwari V., Oh M.J., Kovacs M., Shukla S.Y., Valyi-Nagy T., Shukla D. (2008). Role for nectin-1 in herpes simplex virus 1 entry and spread in human retinal pigment epithelial cells. Febs J..

[16] Gianni T., Massaro R., Campadelli-Fiume G. (2015). Dissociation of HSV gL from gH by alphavbeta6- or alphavbeta8-integrin promotes gH activation and virus entry. Proc. Natl. Acad. Sci. USA.

[17] Manservigi R., Spear P.G., Buchan A. (1977). Cell fusion induced by herpes simplex virus is promoted and suppressed by different viral glycoproteins. Proc. Natl. Acad. Sci. USA.

[18] Compton T., Kurt-Jones E.A., Boehme K.W., Belko J., Latz E., Golenbock D.T., Finberg R.W. (2003). Human cytomegalovirus activates inflammatory cytokine responses via CD14 and Toll-like receptor 2. J. Virol..

[19] Boehme K.W., Guerrero M., Compton T. (2006). Human cytomegalovirus envelope glycoproteins B and H are necessary for TLR2 activation in permissive cells. J. Immunol..

[20] Cai M., Li M., Wang K., Wang S., Lu Q., Yan J., Mossman K.L., Lin R., Zheng C. (2013). The herpes simplex virus 1-encoded envelope glycoprotein B activates NF-kappaB through the Toll-like receptor 2 and MyD88/TRAF6-dependent signaling pathway. PLoS ONE.

[21] Leoni V., Gianni T., Salvioli S., Campadelli-Fiume G. (2012). Herpes simplex virus glycoproteins gH/gL and gB bind Toll-like receptor 2, and soluble gH/gL is sufficient to activate NF-kappaB. J. Virol..

[22] Manservigi R., Grossi M.P., Gualandri R., Balboni P.G., Marchini A., Rotola A., Rimessi P., Di Luca D., Cassai E., Barbanti-Brodano G. (1990). Protection from herpes simplex virus type 1 lethal and latent infections by secreted recombinant glycoprotein B constitutively expressed in human cells with a BK virus episomal vector. J. Virol..

[23] Marino A., Pergolizzi S., Lauriano E.R., Santoro G., Spataro F., Cimino F., Speciale A., Nostro A., Bisignano G. (2015). TLR2 activation in corneal stromal cells by Staphylococcus aureus-induced keratitis. Apmis Acta Pathol. Microbiol. Immunol. Scand..

[24] Goldstein D.A., Mis A.A., Oh F.S., Deschenes J.G. (2009). Persistent pupillary dilation in herpes simplex uveitis. Can. J. Ophthalmol..

[25] Lin P. (2015). Infectious Uveitis. Curr. Ophthalmol. Rep..

[26] De-la-Torre A., Valdes-Camacho J., Foster C.S. (2017). Bilateral Herpes Simplex Uveitis: Review of the Literature and Own Reports. Ocul. Immunol. Inflamm..

[27] Laquerre S., Argnani R., Anderson D.B., Zucchini S., Manservigi R., Glorioso J.C. (1998). Heparan sulfate proteoglycan binding by herpes simplex virus type 1 glycoproteins B and C, which differ in their contributions to virus attachment, penetration, and cell-to-cell spread. J. Virol..

[28] Marconi P., Manservigi R. (2014). Herpes simplex virus growth, preparation, and assay. Methods Mol. Biol..

[29] Revello M.G., Gualandri R., Manservigi R., Gerna G. (1991). Development and Evaluation of an Elisa Using Secreted Recombinant Glycoprotein-B for Determination of IgG Antibody to Herpes-Simplex Virus. J. Virol. Methods.

[30] Cortesi R., Argnani R., Esposito E., Dalpiaz A., Scatturin A., Bortolotti F., Lufino M., Guerrini R., Cavicchioni G., Incorvaia C. (2006). Cationic liposomes as potential carriers for ocular administration of peptides with anti-herpetic activity. Int. J. Pharm..

[31] Blanco A.R., Nostro A., D’Angelo V., D’Arrigo M., Mazzone M.G., Marino A. (2017). Efficacy of a Fixed Combination of Tetracycline, Chloramphenicol, and Colistimethate Sodium for Treatment of Candida albicans Keratitis. Investig. Ophthalmol. Vis. Sci..

[32] Marino A., Santoro G., Spataro F., Lauriano E.R., Pergolizzi S., Cimino F., Speciale A., Nostro A., Bisignano G., Dugo G. (2013). Resveratrol role in Staphylococcus aureus-induced corneal inflammation. Pathog. Dis..

[33] Schmittgen T.D., Livak K.J. (2008). Analyzing real-time PCR data by the comparative C(T) method. Nat. Protoc..

[34] Lauriano E.R., Pergolizzi S., Capillo G., Kuciel M., Alesci A., Faggio C. (2016). Immunohistochemical characterization of Toll-like receptor 2 in gut epithelial cells and macrophages of goldfish Carassius auratus fed with a high-cholesterol diet. Fish Shellfish Immunol..

[35] Lauriano E.R., Silvestri G., Kuciel M., Zuwala K., Zaccone D., Palombieri D., Alesci A., Pergolizzi S. (2014). Immunohistochemical localization of Toll-like receptor 2 in skin Langerhans’ cells of striped dolphin (Stenella coeruleoalba). Tissue Cell.

[36] Revello M.G., Percivalle E., Zannino M., Rossi V., Gerna G. (1991). Development and Evaluation of a Capture Elisa for Igm Antibody to the Human Cytomegalovirus Major DNA-Binding Protein. J. Virol. Methods.

[37] Caselli E., Balboni P.G., Incorvaia C., Argnani R., Parmeggiani F., Cassai E., Manservigi R. (2000). Local and systemic inoculation of DNA or protein gB1s-based vaccines induce a protective immunity against rabbit ocular HSV-1 infection. Vaccine.

[38] Manservigi R., Boero A., Argnani R., Caselli E., Zucchini S., Miriagou V., Mavromara P., Cilli M., Grossi M.P., Balboni P.G. (2005). Immunotherapeutic activity of a recombinant combined gB-gD-gE vaccine against recurrent HSV-2 infections in a guinea pig model. Vaccine.

[39] Cortesi R., Ravani L., Rinaldi F., Marconi P., Drechsler M., Manservigi M., Argnani R., Menegatti E., Esposito E., Manservigi R. (2013). Intranasal immunization in mice with non-ionic surfactants vesicles containing HSV immunogens: A preliminary study as possible vaccine against genital herpes. Int. J. Pharm..

[40] Alekseev O., Tran A.H., Azizkhan-Clifford J. (2012). Ex vivo organotypic corneal model of acute epithelial herpes simplex virus type I infection. J. Vis. Exp. Jove.

[41] Cowdry E.V., Nicholson F.M. (1923). Inclusion Bodies in Experimental Herpetic Infection of Rabbits. J. Exp. Med..

[42] Ljubimov A.V., Saghizadeh M. (2015). Progress in corneal wound healing. Prog. Retin. Eye Res..

[43] Imai T., Arii J., Minowa A., Kakimoto A., Koyanagi N., Kato A., Kawaguchi Y. (2011). Role of the herpes simplex virus 1 Us3 kinase phosphorylation site and endocytosis motifs in the intracellular transport and neurovirulence of envelope glycoprotein B. J. Virol..

[44] Niazy N., Temme S., Bocuk D., Giesen C., Konig A., Temme N., Ziegfeld A., Gregers T.F., Bakke O., Lang T. (2017). Misdirection of endosomal trafficking mediated by herpes simplex virus-encoded glycoprotein B. Faseb J. Off. Publ. Fed. Am. Soc. Exp. Biol..

[45] Ghasemi H., Ghazanfari T., Yaraee R., Faghihzadeh S., Hassan Z.M. (2011). Roles of IL-8 in ocular inflammations: A review. Ocul. Immunol. Inflamm..

[46] Yu Z., Li S., Huang Y.Y., Fong Y., Wong R.J. (2007). Calcium depletion enhances nectin-1 expression and herpes oncolytic therapy of squamous cell carcinoma. Cancer Gene Ther..

[47] Roos J.C., Haridas A.S. (2015). Prolonged mydriasis after inadvertent topical administration of the calcium channel antagonist amlodipine: Implications for glaucoma drug development. Cutan. Ocul. Toxicol..

[48] Nazli A., Kafka J.K., Ferreira V.H., Anipindi V., Mueller K., Osborne B.J., Dizzell S., Chauvin S., Mian M.F., Ouellet M. (2013). HIV-1 gp120 induces TLR2- and TLR4-mediated innate immune activation in human female genital epithelium. J. Immunol..

[49] Krawczyk A., Dirks M., Kasper M., Buch A., Dittmer U., Giebel B., Wildschutz L., Busch M., Goergens A., Schneweis K.E. (2015). Prevention of herpes simplex virus induced stromal keratitis by a glycoprotein B-specific monoclonal antibody. PLoS ONE.

[50] Khan A.A., Srivastava R., Spencer D., Garg S., Fremgen D., Vahed H., Lopes P.P., Pham T.T., Hewett C., Kuang J. (2015). Phenotypic and functional characterization of herpes simplex virus glycoprotein B epitope-specific effector and memory CD8+ T cells from symptomatic and asymptomatic individuals with ocular herpes. J. Virol..

[51] Henrick B.M., Yao X.D., Taha A.Y., German J.B., Rosenthal K.L. (2016). Insights into Soluble Toll-Like Receptor 2 as a Downregulator of Virally Induced Inflammation. Front. Immunol..

